# Recent Understandings of Biology, Prophylaxis and Treatment Strategies for Hypertrophic Scars and Keloids

**DOI:** 10.3390/ijms19030711

**Published:** 2018-03-02

**Authors:** Ho Jun Lee, Yong Ju Jang

**Affiliations:** 1 Department of Otorhinolaryngology-Head and Neck Surgery, Chuncheon Sacred Heart Hospital, College of Medicine, Hallym University, Chuncheon 24253, Korea; leehj@hallym.or.kr; 2 Department of Otolaryngology, Asan Medical Center, University of Ulsan College of Medicine, Seoul 05505, Korea

**Keywords:** keloid, hypertrophic scar, scar biology, scar prevention, scar treatment

## Abstract

Hypertrophic scars and keloids are fibroproliferative disorders that may arise after any deep cutaneous injury caused by trauma, burns, surgery, etc. Hypertrophic scars and keloids are cosmetically problematic, and in combination with functional problems such as contractures and subjective symptoms including pruritus, these significantly affect patients’ quality of life. There have been many studies on hypertrophic scars and keloids; but the mechanisms underlying scar formation have not yet been well established, and prophylactic and treatment strategies remain unsatisfactory. In this review, the authors introduce and summarize classical concepts surrounding wound healing and review recent understandings of the biology, prevention and treatment strategies for hypertrophic scars and keloids.

## 1. Introduction

Many life situations result in injury to the skin. Physical trauma, surgical incisions, burn injuries, vaccinations, skin piercings, herpes infection and even insect bites can cause skin injury and resultant scar problems. Each year in the developed world, approximately 100 million people suffer from scar-related issues [1]. Most superficial injuries do not leave significant scars, but deep cutaneous injuries occasionally produce serious problems, hypertrophic scars and keloids [2]. Cosmetic problems, functional problems such as contractures and patients’ subjective symptoms including pruritus and pain can cause hypertrophic scars and keloids to dramatically affect patients’ quality of life, physical status and psychological health [3]. Hypertrophic scars and keloids are fibroproliferative disorders that result from abnormal wound healing, defined as increased or decreased regulation of certain wound healing processes. Understanding the major mechanisms underlying abnormal wound healing and correcting them will benefit numerous patients, like the wide-spread public health effects of antibiotics in the twentieth century. Many studies on hypertrophic scars and keloids have been reported, and our understanding of these conditions is improving. However, the pathophysiology remains extremely complex. In this review, we introduce and summarize the classical concepts of wound healing and review the recent biological advances in treatment, as well as the manner in which these advances translate into preventive and treatment strategies for hypertrophic scars and keloids. This review included the basic knowledge on scar biology any kind of physician should know and may be appropriate for general physicians rather than scar specialists.

## 2. Methods

**Figure 1:**
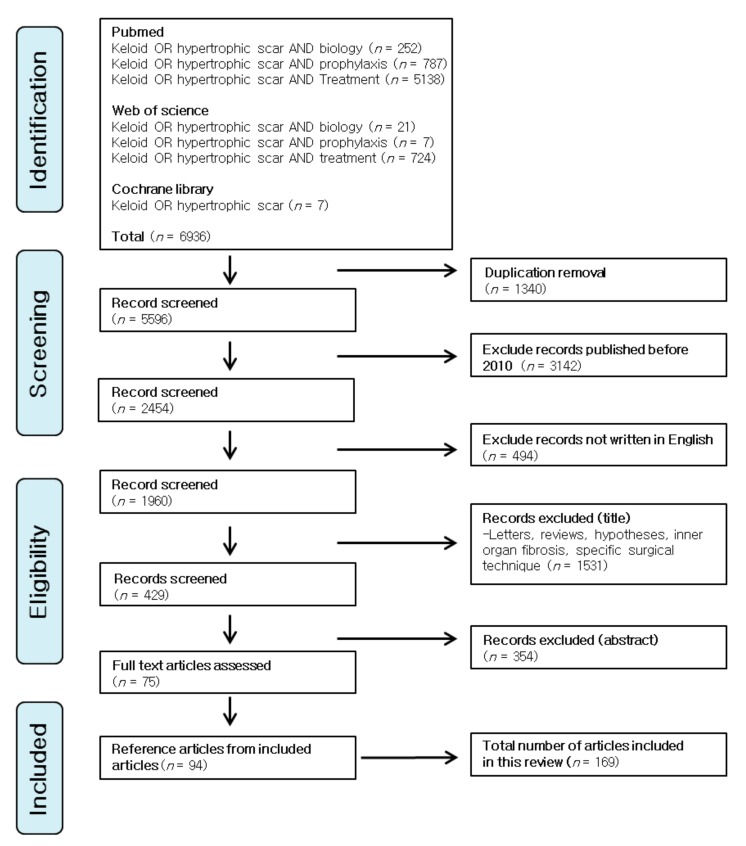
The flowchart of the literature search for this review.

The original articles dealing with the biology, prophylaxis and treatment strategies for hypertrophic scars and keloids were searched and reviewed. PubMed, Web of Science and Cochrane library databases were searched on the keywords: hypertrophic scar OR keloid AND biology; hypertrophic scar OR keloid AND prophylaxis; hypertrophic scar OR keloid AND treatment. Time limits were from 1 January 2010 to the present. In addition, important reference articles from the included articles were also reviewed. Several meta-analysis were also reviewed to estimate the outcome of a certain treatment modality. Duplicates, letters, reviews, hypotheses articles dealing with the fibrotic disorders on internal organs, studies dealing with specific surgical techniques and studies published in a language other than English were excluded. Figure 1 shows the flowchart of the literature search for this review.

## 3. Classical Concepts of Wound Healing

The classical model of wound healing involves three distinct, but overlapping phases that follow a time sequence: the inflammatory phase, the proliferative phase and the remodeling phase. The first phase of wound healing is the inflammatory phase that starts immediately after tissue injury and lasts for approximately 2–3 days after injury. Coagulation cascades, complement activation and platelet degranulation prevent further fluid and blood losses by creating platelet plugs and a fibrin matrix [4]. The immune system and inflammatory reactions are activated to prevent infection and removing devitalized tissues [5]. Neutrophils are recruited by chemotactic factors produced by platelet and bacterial degranulations [6], and monocytes are recruited and differentiated into macrophages 2–3 days after injury.

The second phase of wound healing is the proliferative phase. This phase of new tissue formation occurs approximately 2–3 days after tissue damage and may last for 3–6 weeks. Active cellular proliferation and migration characterize this phase. Keratinocytes migrate to the damaged dermis; new blood vessels grow inward within the damaged tissue; and new capillaries replace the fibrin matrix with granulation tissue via the actions of macrophages and fibroblasts. Granulation tissue forms a new substrate for keratinocyte migration. Keratinocytes proliferate and mature within granulation tissue along the wound margin, restoring the protective function of the epithelium. In the late proliferative phase, a portion of the fibroblasts differentiates into myofibroblasts in association with macrophages. Fibroblasts and myofibroblasts produce extracellular matrix (ECM), mainly in the form of collagen; this accumulated collagen forms most of the eventual scar [7]. Other constituents of ECM include elastin, hyaluronic acids and proteoglycans. Myofibroblasts, which contain actin filaments, have contractile properties and help bring the edges of the wound together over time [8]. Once wound closure is accomplished, the final remodeling phase commences. This phase is characterized by degradation of excessive tissue, transforming immature healing products into a mature form. Remodeling may last for a year or more. Excessive ECM is degraded and remodeled from type III collagen, the main component of ECM present during the early wound healing process, to mature type I collagen.

## 4. Important Proteins and Cytokines in the Wound Healing Processes

**Figure 2:**
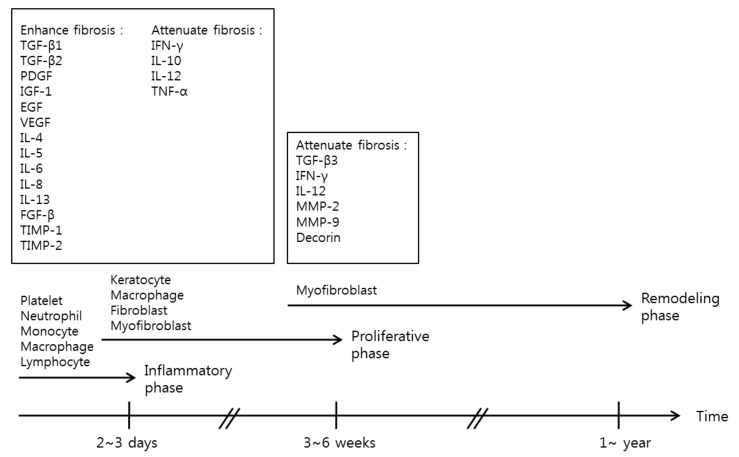
Important proteins and cytokines in the wound healing processes. The classical model of wound healing involves three distinct, but overlapping phases that follow a time sequence: the inflammatory, proliferative and remodeling phases. Important cells, proteins and cytokines in each phase are listed.

It is important to achieve a proper balance between these wound healing phases. Synthesis and degradation of ECM should be balanced, otherwise wound healing may be delayed or result in excessive scars. Important proteins and cytokines that influence balanced wound healing processes are summarized herein (Figure 2) and are important for understanding current investigations in keloid treatment.

### 4.1. Inflammatory and Proliferative Phase

Prolonged and excessive inflammatory reactions result within the context of increased fibroblast activity, which in turn produces excessive ECM. In this phase, degranulation of platelets releases and activates transforming growth factor β (TGF-β), particularly TGF-β1, TGF-β2, platelet-derived growth factor (PDGF), insulin-like growth factor (IGF-1) and epidermal growth factor (EGF). Vascular endothelial growth factor (VEGF), which is produced by epidermal cells, is a positive regulator of angiogenesis. Because of this, overexpression of VEGF is related to excessive capillary formation, collagen type I production and overall scar volume increase [9]. These cytokines are not only fibrogenic growth factors, but also chemotactic agents for epithelial cells, endothelial cells, neutrophils, macrophages, mast cells and fibroblasts [4,8]. Fibroblasts originating in keloid tissues show increased receptors to these growth factors and demonstrate increased responsiveness compared with fibroblasts from normal tissues [10,11,12,13]. Tissue inhibitors of metalloproteinases (TIMPs) are endogenous inhibitors of matrix metalloproteinases (MMPs); thus, increased levels of TIMPs, especially TIMP-1 and TIMP-2, are associated with hypertrophic scar formation [9]. Tumor necrosis factor-α (TNF-α) is an inflammatory cytokine produced by monocytes and macrophages during the inflammatory phase. It has been known that this cytokine induces collagen degranulation and contributes to minimizing excessive scarring. One suggested mechanism is that TNF-α increases the MMP1/TIMP3, MMP2/TIMP3 ratios [14]. However, other studies showed that the biologic effect of TNF-α was not the same on the fibroblasts from lung and skin tissues showing tissue specificity [15] and TNF-α induced epithelial-mesenchymal transition in human skin wound healing [16]. Therefore, it is still unclear whether TNF-α would promote or attenuate scar formation.

Immune responses are also related to wound healing processes. T helper CD4 cells are thought to be major immunoregulatory cells during wound healing processes. CD4 T cells express Th1 or Th2 responses [17]. Th1 responses produce interferon-γ and interleukin (IL)-12 and are thought to be related to the attenuation of fibrogenesis. Th2 responses of CD4 cells are generally likely related to fibrogenesis. IL-4, IL-5, IL-6 and IL-13 are thought to be related to pro-fibrosis [18,19], but IL-10 is thought to be related to anti-fibrosis [20,21,22]. These cytokines are essential for promoting or impeding the fibroblast recruitment and proliferation, ECM deposition, angiogenesis and re-epithelialization [4]. Endothelial cytokines including IL-8, IGF-1, fibroblast growth factor (FGF)-β and heparin promote angiogenesis. Wound re-epithelialization is enhanced by EGF, TGF-α and IGF-1 [4].

### 4.2. Remodeling Phase

During the remodeling phase, excessive ECM is degraded, and collagen type III, an immature collagen form, is converted to mature collagen type I. TGF-β3 is considered to play a role in reducing the newly-synthesized ECM [23]. Significantly lower TGF-β3 mRNA expression was found in keloid tissues [24,25]. However, TGF-β isoforms (TGF-β1, TGF-β2 and TGF-β3) do not present its activity as isolated ligands, but are also associated with receptors and activity modulators; therefore, the mere presence or absence of TGF-β may not fully explain abnormal wound healing [26]. Members of the MMP family have major effects on ECM degradation and remodeling and mediate the degradation of type III and type I collagens, the major components of ECM [27,28]. MMP-2 and MMP-9 are active during the remodeling phase. MMP-9 is involved in degradation of type IV and V collagens, fibronectin and elastin. MMP-2 plays an important role in ECM remodeling by degrading denatured collagen [29,30]. MMPs have a downregulating effect on inflammation by decreasing and antagonizing chemokines [31,32]. Immunity, cell migration and angiogenesis are also influenced by MMPs [33]. MMP activities are regulated by TIMPs. Decorin is a proteoglycan component of dermal connective tissue that binds to type I collagen fibrils and influences TGF-β [34]. This protein is decreased in keloids and hypertrophic scars [35]. By binding and neutralizing TGF-β, decorin decreases the stimulatory effects of TGF-β on collagen, fibronectin and glycosaminoglycan synthesis [17]. Decorin also inhibits angiogenesis by interacting with VEGF receptors (VEGFR2) and by inhibiting hepatocyte growth factors and PDGF [36]. Decorin’s antifibrotic properties are receiving attention as a future therapeutic agent [37,38].

## 5. Recent Findings of Scar Biology

Here, we introduce some recent findings on scar biology. These consist of some factors that influence pro-fibrotic or anti-fibrotic pathways.

### 5.1. Hypoxia

Oxygen has long been known to be an important factor in wound healing [39,40]. There have been many reports suggesting that a hypoxic environment is associated with keloid formation [41,42]. Zhao et al. measured the quantity of hypoxia inducible factor (HIF)-1α in keloid and normal tissues and reported that keloid tissues are relatively hypoxic tissues compared to normoxic tissues, and hypoxia induces a pro-fibrotic state in dermal fibroblasts via the TGF-β1/SMAD3 pathway [43].

### 5.2. Periostin

Periostin is a secreted extracellular matrix (ECM) protein, which was originally identified in osteoblast, periodontal ligament and periosteum [44]. This matricellular protein is expressed in the basement membrane, dermis and hair follicle [45]. Periostin is induced by TGF-β in human dermal fibroblast and has an important role in wound healing and scar pathogenesis by inducing angiogenesis, fibroblast proliferation and myofibroblast persistence [45,46,47]. It starts to increase its expression from a few days after injury, peaking after about seven days after injury and decreasing afterwards [48,49]. Many authors have reported that periostin is abnormally elevated in hypertrophic scars and keloids compared to normal tissues [45,46,50,51] and implicates periostin as a possible therapeutic target in the treatment of hypertrophic scars and keloid.

### 5.3. MicroRNAs

MicroRNAs (miRNAs) are a group of short noncoding RNAs that pair complementarily with target genes and silence that genes post-transcriptionally. It thereby regulates negatively the expression of their target genes. miRNAs are thought to be deregulated in many skin diseases such as malignant skin diseases and keloids [52,53,54,55]. Some researchers performed miRNA expression microarrays in keloids and normal tissues [55,56] and reported upregulated or downregulated miRNAs in keloid tissues compared to normal tissues. *miRNA-199a-5p* [57], *miRNA-21* [58,59,60,61], *miRNA-146a* [62], *miRNA-1224-5p* [56], *miRNA-31* [63], and so forth, were investigated and showed potential in the treatment of hypertrophic scars and keloids.

## 6. Preventions and Treatment Strategies for Hypertrophic Scars and Keloids

Because the processes are so complicated, the definitive processes that underlie excessive scar formation are yet to be elucidated. So far, preventions and treatment strategies mainly focus on reducing inflammation. Other therapies, targeting genes and molecules, require more study prior to being introduced in clinical practice. The current treatment strategies for hypertrophic scars and keloids are listed below and summarized in Table 1.

### 6.1. Prevention

#### 6.1.1. Tension-Free Primary Closure

Regardless of a patient’s tendency to exhibit bad scars (or not), (1) debridement of inviable or severely contaminated tissues, (2) adequate hemostasis to prevent hematoma, seroma or abscess formation and (3) rapid primary closure using tension-free techniques are wound care basics and are very important for minimizing the effects of bad scars. Wound epithelialization that is delayed beyond 10–14 days increases the risk of hypertrophic scars, and quick primary closure to induce rapid epithelialization is necessary to achieve good scarring [64]. The importance of tension-free closure techniques cannot be overstated. Wounds that are subject to tension tend to develop into bad scars [65]. The exact molecular mechanisms that govern how our skin responds to physical tension remain uncertain; however, several pathways that convert mechanical forces into biochemical responses have been investigated and reported. This process is called mechanotransduction [66]. Gurtner et al. reported on the fibrotic effects of mechanical tension and described the preventive effect of offloading wound tension on scar formation [67].

#### 6.1.2. Passive Mechanical Stabilization

To prevent wound stretching and consequential mechanotransduction, prolonged passive mechanical wound stabilization has been applied [68,69,70,71] using paper tapes or silicone sheets. Paper tapes help alleviate scar formation, and silicone sheeting is superior to paper tapes because it avoids repeated epidermal avulsion.

Other mechanisms of silicone sheets include occlusion and hydration of the scar surface. The inherent antifibrotic properties of silicone are not definite [72]. Silicone sheeting is recommended for use from two weeks after primary wound treatment for more than 12 h a day for at least two months. For body areas where silicone sheets do not easily fit, silicone gel can be applied.

#### 6.1.3. Flavonoids

Flavonoids (or bioflavonoids) are naturally-derived substances from various plants and have been used for preventing severe scar formation. Several studies have reported the efficacy of flavonoid scar gels like Contractubex Gel (Merz Pharma, Frankfurt, Germany) or Mederma Skin Care Gel (Merz Pharmaceuticals, Greensboro, NC, USA). The efficacy of these gel products is controversial [73,74,75,76,77], but other flavonoids like quercetin exert antifibrotic actions. These actions may be mediated through induction of MMP-1 or inhibition of SMAD2, SMAD3 or SMAD4 expression [77,78]. The instructions of flavonoids, for instance, Contractubex Gel, is as follows: (1) start two weeks after primary wound treatment; and (2) twice daily for four to six months.

#### 6.1.4. Pressure Therapy

Cutaneous wound compression has been used not only for prevention, but also for treatment of hypertrophic scars and keloids. Although pressure therapy reduces the subjective and objective signs and symptoms of hypertrophic scars and keloids, the scientific evidence supporting their use is weak, and their clinical efficacy is also controversial [79]. The suggested mechanisms underlying pressure therapy include occlusion of blood vessels and limiting the delivery of inflammatory cytokines, nutrients and oxygen from blood vessels to scar tissue [80,81,82,83,84]. Increasing apoptosis may be another mechanism of pressure therapy [85]. There are no comparative analyses of pressure amount, and the pressure amount that is used clinically relies on empirical reports. Currently, the recommended amount is 15–40 mm Hg for more than 23 h a day for at least six months [83,86].

### 6.2. Current Treatment Strategies

#### 6.2.1. Corticosteroids

Intralesional steroid injection, steroid tapes/plasters and steroid ointments have been used to treat hypertrophic scars and keloids. Intralesional injection is the most popular method for steroid administration, although steroid tapes/plasters are gaining popularity [87]. The mechanism underlying this therapy is attributed to its anti-inflammatory effect [72]. In addition, steroid therapy seems to reduce collagen synthesis, glycosaminoglycan production, fibroblast proliferation and degeneration of collagen and fibroblasts [88,89]. Another suggested mechanism is induction of vasoconstriction mediated by binding of the topical steroid to classical glucocorticoid receptors [2]. Resolution rates for keloids treated with intralesional steroid injections are variable and range from 50% to 100% and recur in 9% to 50% [90]. Most previous studies used triamcinolone acetonide (TAC), injected alone or in combination with other treatment modalities such as 5-FU, verapamil, cryotherapy or surgery. The concentrations of injectable TAC vary from 10 to 40 mg/mL, but the recommended concentration of TAC in monotherapy is 40 mg/mL for keloid resolution [91]. The injection is performed 1–2 times a months until the scar has flattened. Intralesional steroid injections could cause side effects such as skin atrophy or telangiectasia.

#### 6.2.2. Scar Revision Surgery

Surgical excision is a traditional treatment for hypertrophic scars and keloids. The remodeling phase of classical wound healing may last for more than one year; therefore, excision of hypertrophic scars or keloids should be considered after at least one year of primary wound treatment therapy. As time goes by, hypertrophic scars tend to regress naturally or with conservative treatment such as steroid injections. Therefore, in many cases, there is no need to perform scar revision surgery. For keloids, surgical excision alone frequently results in disappointing outcomes. To improve postoperative surgical outcomes, multimodal combination therapy such as postoperative steroid application or radiotherapy might be added. When surgeons perform scar revision surgery, they should establish tension-free wound closure in order to decrease tension-related inflammation and thereby reduce recurrence. Various techniques including three-layered sutures, subcutaneous/fascial tensile reduction sutures, Z-plastics or local flap reconstruction can be utilized on a case-by-case basis [92,93]. Recurrence rates of hypertrophic scars after scar revision surgery are low, but the recurrence rate of keloids after scar revision surgery is 45% to 100% [94,95,96].

#### 6.2.3. Cryotherapy

Cryotherapy has been used to treat hypertrophic scars or keloids as a monotherapy or in conjunction with other therapies such as intralesional steroid injections [97]. Treatments that combine cryotherapy and intralesional triamcinolone injections significantly improve hypertrophic scars and keloids [98,99,100]. Delivery methods for cryotherapy are variable and include sprays, contact or the intralesional-needle cryoprobe method. The intralesional-needle cryoprobe method shows better results than the spray or contact method, producing rapid re-epithelialization [101]. The suggested mechanism underlying cryotherapy is tissue necrosis induced by vascular damage. It seems that necrotized tissues induced by frostbite (as opposed to burn injury) secrete unique inflammatory cytokines; therefore, the responses of fibroblasts may differ [2]. Cryotherapy success rates range from 32 to 74% after several sessions [102,103,104].

#### 6.2.4. Radiotherapy

Several studies have shown the effectiveness of radiotherapy on keloid treatment. Both external beam therapy and brachytherapy (or internal radiation therapy) have been used and studied for treatment of keloids. Radiotherapy is generally conducted as an adjuvant treatment 24 to 48 h after scar revision surgery, and the recommend radiation dose is 40 Gray over several divided sessions to minimize adverse effects [105]. The suggested mechanism of radiotherapy for treating keloids is anti-angiogenesis and successive anti-fibroblast activity. Suppression of angiogenesis decreases delivery of inflammatory cytokines, and successive inhibition of fibroblast activity results in decreased collagen synthesis, thus suppressing keloid development [106,107]. Radiotherapy carries an inherent risk of carcinogenesis; therefore, even though the risk is low [108,109], radiation-vulnerable areas, including the thyroid and breast, should be treated after achieving informed consent and with abundant cautions. Shen et al. reported the recurrence rate of 9.59% [109]. Recently, radioactive skin patches have been used for localized skin diseases like skin cancers or keloids [110,111]. Radioactive skin patches use various kinds of radionuclides and have variable effectiveness for treating keloids. These patches are frequently used in combination with other available treatment.

#### 6.2.5. Laser Therapy

Laser therapy was introduced for keloid treatment in the 1980s [112], and several kinds of lasers with various wavelengths were investigated and reported. Among these, the most popular laser used to treat hypertrophic scars and keloids is the 585-nm pulsed dye laser (PDL) [113]. The recommended energy is 6.0 to 7.5 J/cm^2^ (7-mm spot) or 4.5 to 5.5 J/cm^2^ (10-mm spot) [114], and two to six sessions of treatment may be needed [113]. The 1064-nm Nd:YAG laser is another popular laser for treating hypertrophic scars and keloids. For this laser, the recommended energy is 14 J/cm^2^ (5-mm spot), with the procedure being repeated every three to four weeks [115,116]. These laser treatments vaporize blood vessels. By doing this, inflammatory cytokines are limited in their ability to reach hypertrophic scars and keloids, thereby suppressing the development of aberrant scars. Possible side effects of laser therapy include hyperpigmentation, hypopigmentation, blister formation and postoperative purpura [117,118,119,120].

#### 6.2.6. 5-Fluorouracil

5-FU is a medication mainly used to treat cancer. By injecting it into a vein, it can be used for the treatment of esophageal, stomach, pancreatic, colon, breast and cervical cancers. It can also be used topically for actinic keratosis and basal cell carcinoma in a cream or solution formulation [87]. 5-FU has also been used to treat keloids [121]. The suggested mechanism is anti-angiogenesis, anti-fibroblast proliferation and anti-collagen Type I expression induced by TGF-β [122,123,124]. This therapy is used solely or in combination with another treatment, and intralesional injection is the preferred method of delivery. Nanda et al. reported scar size reduction in a majority of patients in whom 5-FU was injected intralesionally weekly for 12 weeks in a concentration of 50 mg/mL [122]. Possible side effects include pain and ulceration. A systematic review reported 45% to 96% of effectiveness [125].

### 6.3. Emerging Therapies

#### 6.3.1. Mesenchymal Stem Cell Therapy

Mesenchymal stem cells (MSCs) have immunomodulatory and antifibrotic effects by secreting paracrine growth factors [126,127,128,129]. The antifibrotic effects of MSC on fibrotic diseases such as myocardial infarctions, renal fibrosis or liver cirrhosis have been investigated and reported [130,131,132,133,134,135,136]. MSCs are also used to prevent or attenuate excessive inflammatory processes that are characteristic of hypertrophic scars and keloids. MSC treatments have variable delivery methods and doses [137]. Delivery is conducted via systemic injections, local injections (at the wound, intradermal or subcutaneously) or via an engineered MSC-seeded tissue scaffold [138,139,140,141]. The possible mechanisms underlying MSC treatment include: (1) modulation and inhibition of proinflammatory cell activity; (2) antifibrotic activity via downregulation of myofibroblast differentiation and collagen type I and III production; and (3) promotion of normal angiogenetic activity that aids in normal wound healing [137,142]. Even though many researchers have reported anti-inflammatory and anti-fibrotic effects of MSC, there are reports of possible proinflammatory actions of MSC [143,144,145]. More investigations and long-term preclinical studies should be conducted to apply this method to clinical practice.

#### 6.3.2. Fat Grafting

Autologous fat grafting or lipotransfer, underneath or into the wound, has been performed for patients with hypertrophic scars or keloids. Several studies have reported the effectiveness of fat grafting on severely-scarred lesions [146,147,148]. These reports showed beneficial effects on excessive scar lesions, and side effects were rarely reported. The mechanism underlying fat injections is believed to be that transferred fat tissues deliver adipose-tissue derived MSCs to the wound.

#### 6.3.3. Interferon

Interferon (IFN) is comprised of cytokines that have anti-proliferative and anti-fibrotic effects. As mentioned earlier, IFNs attenuate collagen synthesis and fibroblast proliferation by downregulating TGF-β1. Although adverse effects including pain at the injection site and flu-like symptoms are relatively common in IFN treatment, some authors reported a good outcome of combination therapy of IFN α-2b with TAC injection [149,150].

#### 6.3.4. Transforming Growth Factor-β

TGF-β isoforms (TGF-β1,2,3) had long been a target of anti-keloid therapy. Several studies showed that the ratio of TGF-β3 and TFG-β1 and 2 is important in scar progression or remission [151,152]. Many studies had been performed to investigate the effect of exogenous TGF-β1 and 2 neutralizing antibodies and exogenous TFG-β3 and had proven the effect of TGF-β isoforms; TGF-β1 and 2 increase fibrosis, and TGF-β3 attenuates fibrosis [153]. Recombinant human TGF-β3, avotermin (planned trade name Juvista) showed successful results in phase I/II clinical trials [154,155,156], but failed in phase III clinical trials.

#### 6.3.5. Botulinum Toxin A

Botulinum toxin, which is derived from *Clostridium botulinum*, is a potent neurotoxin that blocks neuromuscular transmission. Some authors have reported that botulinum toxin type A can minimize scar formation by reducing muscle tension during wound healing, causing the fibroblast cell cycle to be paused in a non-proliferative state, G0 or G1, and influencing TGF-β1 expression [157,158,159,160,161]. Intralesional injection was the preferred delivery method, and 70–140 U of Type A botulinum toxin was delivered per sessions at one- or three-month intervals for three or nine months (three sessions) [160,162,163,164]. Treatment outcomes were generally favorable, and patient satisfaction was high. Improvement was also reported regarding pain, tenderness and itching sensation [160,162,163].

#### 6.3.6. Bleomycin

Bleomycin is a cytotoxic, antineoplastic, antiviral and antibacterial agent [165], derived from *Streptomyces verticillus*, and has been used for dermatologic diseases such as warts. This agent has also been used for hypertrophic scars and keloids. Several studies have found that bleomycin-treated human dermal fibroblasts showed diminished collagen synthesis, even with the co-existence of TFG-β1, and a reduction in the levels of lysyl-oxidase, which is involved in the maturation of collagen. In addition, apoptosis was also induced by bleomycin treatment [166,167,168,169]. Intralesional injection is the preferred delivery method, and 1.5 IU/mL of bleomycin were injected two to six sessions at monthly intervals. Several studies reported that complete flattening was achieved in 54% to 73% of keloid patients [166,167] and other symptoms like itching and pain were also resolved. Possible side effects include injection site pain, ulceration, atrophy and hyperpigmentation, but systemic side effects were not observed [165,167].

## 7. Conclusions

Hypertrophic scars and keloids result from abnormal wound healing. Excessive ECM deposition is characteristic of these lesions. Increased inflammatory and proliferative processes and decreased remodeling processes cause excessive ECM deposition. Genetic and systemic factors are also related to these excessively scarring lesions. Although encouraging results of molecular- or cytokine-targeting therapies are being continuously reported, current prophylaxis and treatment strategies still mainly focus on decreasing inflammatory processes. Further understanding of the mechanisms underlying excessive scarring is needed to develop more effective prophylaxis and treatment strategies.

## Figures and Tables

**Table 1:** Current treatment strategies for hypertrophic scars and keloids.

Categories	Modalities	Suggested Mechanisms	Use
Prophylaxis	Tension-free closure	-Reduce inflammation by reducing mechanotransduction	-Debridement of inviable tissues, adequate hemostasis -Rapid tension free primary closure
	Taping or silicone sheeting	-Reduce inflammation by reducing mechanotransduction: occlusion and hydration	-Start 2 weeks after primary wound treatment -12 h a day for at least 2 months
	Flavonoids	-Induction of MMPs -Inhibition of SMADs expression	-Start 2 weeks after primary wound treatment -Generally twice daily for 4 to 6 months
	Pressure therapy	-Occlusion of blood vessels -Inducing apoptosis	-Pressure of 15 to 40 mmHg -More than 23 h a day for at least 6 months
Treatment (current)	Corticosteroids	-Reducing inflammation and proliferation -Vasoconstriction	-Intralesional injection: triamcinolone 10 to 40 mg/mL -1 to 2 sessions a month (2 to 3 sessions, but can be extended) -Tapes/plasters, ointments are available -Combination is common
	Scar revision	-Direct reduction of scar volume	-At least 1 year after primary wound treatment -Combination is recommended
	Cryotherapy	-Scar tissue necrosis	-Deliver liquid nitrogen using spray, contact or intralesional needle cryoprobe -10 to 20 s freeze-thaw cycles -Combination is common
	Radiotherapy	-Anti-angiogenesis -Anti-inflammation	-Adjuvant after scar revision -24–48 h after scar revision surgery -Total of 40 Gray or less, over several divided sessions
	Laser therapy	-Vaporize blood vessel -Anti-inflammation	-585-nm pulsed dye laser: 6.0–7.5 J/cm^2^ (7 mm spot) or 4.5–5.5 J/cm^2^ (10 mm spot) -1064-nm Nd:YAG laser: 14 J/cm^2^ (5 mm spot) -2 to 6 sessions, every 3–4 weeks
	5-Fluorouracil	-Anti-angiogenesis -Anti-inflammation	-Intralesional injection: 50 mg/mL -Weekly for 12 weeks -Combination is common
Treatment (Emerging)	MSC * therapy	-Modulation of proinflammatory cell activity -Anti-fibrosis -Promote normal angiogenetic activity	-Systemic injection -Local injection (at the wound) -Engineered MSC-seeded tissue scaffold
	Fat grafting	-Deliver adipose-tissue derived MSCs	-Fat injection or fat tissue grafting underneath or into the wound
	Interferon	-Downregulating TGF-β1 -Attenuates collagen synthesis and fibroblast proliferation	-Intralesional injection: 1.5 × 10^6^ IU, twice daily over 4 days
	Human recombinant TGF-β3/TGF-β1 or 2 neutralizing antibody	-Adjust TGF-β3: TGF-β1 or 2 ratio	Not available currently
	Botulinum toxin type A	-Reduce muscle tension during wound healing -Arrest cell cycle in non-proliferative stage -Influence TGF-β1 expression	-Intralesional injection: 70~140 U, 1 or 3 months interval, 3 sessions
	Bleomycin	-Decreasing collagen synthesis -Reduce lysyl-oxidase levels -Induce apoptosis	-Intralesional injection: 1.5 IU/mL, 2 to 6 sessions at monthly interval

* MSC: mesenchymal stem cell; MMPs: matrix metalloproteinases; TGF: transforming growth factor.
